# *N*-(2,4-Dichloro­phen­yl)benzamide

**DOI:** 10.1107/S1600536808012385

**Published:** 2008-05-03

**Authors:** B. Thimme Gowda, Miroslav Tokarčík, Jozef Kožíšek, B. P. Sowmya, Hartmut Fuess

**Affiliations:** a Department of Chemistry, Mangalore University, Mangalagangotri 574 199, Mangalore, India; b Faculty of Chemical and Food Technology, Slovak Technical University, Radlinského 9, SK-812 37 Bratislava, Slovak Republic; c Institute of Materials Science, Darmstadt University of Technology, Petersenstrasse 23, D-64287 Darmstadt, Germany

## Abstract

The conformations of the N—H and C=O bonds in the structure of the title compound, C_13_H_9_Cl_2_NO, are *anti* to each other, similar to that observed in *N*-phenyl­benzamide, *N*-(2-chloro­phen­yl)benzamide, *N*-(4-chloro­phen­yl)benzamide, *N*-(2,3-dichloro­phen­yl)benzamide, *N*-(2,6-dichloro­phen­yl)benzamide and other benzanilides. The amide –NHCO– group forms a dihedral angle of 33.0 (2)° with the benzoyl ring, while the rings are almost coplanar, making a dihedral angle of 2.6 (2)°). The mol­ecules are linked by N—H⋯O hydrogen bonds into infinite chains running along the *b* axis.

## Related literature

For related literature, see: Gowda *et al.* (2003 ▶, 2007*a* ▶,*b* ▶, 2008*a* ▶,*b* ▶).
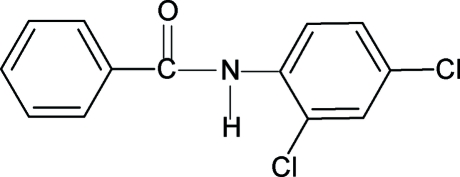


## Experimental

### 

#### Crystal data

C_13_H_9_Cl_2_NO
*M* *_r_* = 266.11
Monoclinic, 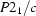

*a* = 11.7388 (6) Å
*b* = 4.7475 (2) Å
*c* = 22.8630 (11) Å
β = 106.360 (4)°
*V* = 1222.56 (10) Å^3^
*Z* = 4
Mo *K*α radiation
μ = 0.51 mm^−1^
*T* = 295 (2) K
0.33 × 0.06 × 0.03 mm

#### Data collection

Oxford Diffraction Xcalibur diffractometer
Absorption correction: analytical [*CrysAlis RED* (Oxford Diffraction, 2007 ▶), based on expressions derived by Clark & Reid (1995 ▶)] *T*_min_ = 0.905, *T*_max_ = 0.987
11465 measured reflections
2311 independent reflections
1209 reflections with *I* > 2σ(*I*)
*R*_int_ = 0.060

#### Refinement

*R*[*F* ^2^ > 2σ(*F* ^2^)] = 0.037
*wR*(*F* ^2^) = 0.089
*S* = 1.06
2311 reflections
157 parameters
1 restraint
H atoms treated by a mixture of independent and constrained refinement
Δρ_max_ = 0.21 e Å^−3^
Δρ_min_ = −0.16 e Å^−3^

### 

Data collection: *CrysAlis CCD* (Oxford Diffraction, 2007 ▶); cell refinement: *CrysAlis RED* (Oxford Diffraction, 2007 ▶); data reduction: *CrysAlis RED*; program(s) used to solve structure: *SHELXS97* (Sheldrick, 2008 ▶); program(s) used to refine structure: *SHELXL97* (Sheldrick, 2008 ▶); molecular graphics: *ORTEP-3* (Farrugia, 1997 ▶) and *DIAMOND* (Brandenburg, 2002 ▶); software used to prepare material for publication: *SHELXL97*, *PLATON* (Spek, 2003 ▶) and *WinGX* (Farrugia, 1999 ▶).

## Supplementary Material

Crystal structure: contains datablocks I, global. DOI: 10.1107/S1600536808012385/om2232sup1.cif

Structure factors: contains datablocks I. DOI: 10.1107/S1600536808012385/om2232Isup2.hkl

Additional supplementary materials:  crystallographic information; 3D view; checkCIF report

## Figures and Tables

**Table 1:** Hydrogen-bond geometry (Å, °)

*D*—H⋯*A*	*D*—H	H⋯*A*	*D*⋯*A*	*D*—H⋯*A*
N1—H1*N*⋯O1^i^	0.805 (16)	2.178 (19)	2.899 (2)	149 (2)

Symmetry code: (i) 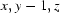.

**Fig. 1.**
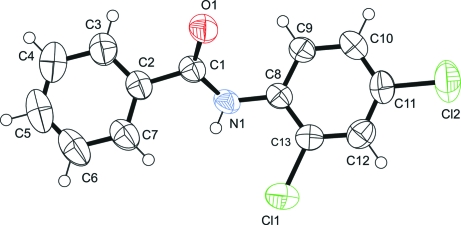
Molecular structure of the title compound showing the atom labelling scheme. Displacement ellipsoids are drawn at the 50% probability level.

**Fig. 2.**
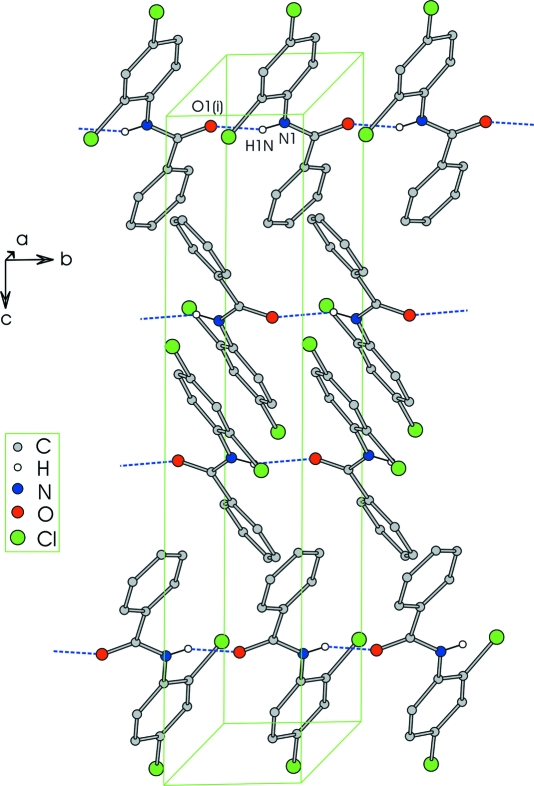
Part of the crystal structure of the title compound with infinite molecular chains running in the [010] direction. The chains are generated by N—H···O(i) hydrogen bonds.[Symmetry operation (i): x,y - 1,z]. H atoms not involved in intermolecular bonding have been omitted.

## supplementary crystallographic information

### Comment

In the present work, the structure of *N*-(2,4-dichlorophenyl)-benzamide (N24DCPBA) has been determined to study the effect of substituents on the structures of benzanilides (Gowda *et al.*, 2003, 2007a,b, 2008a,b). The conformations of the N—H and C=O bonds in the structure of N24DCPBA (Fig.1) are anti to each other, similar to that observed in *N*-(phenyl)-benzamide (NPBA) (Gowda *et al.*, 2003), *N*-(2-chlorophenyl)-benzamide (N2CPBA), *N*-(4-chlorophenyl)-benzamide (N4CPBA), *N*-(2,3-dichlorophenyl)-benzamide(N23DCPBA), *N*-(2,6-dichlorophenyl)-benzamide (N26DCPBA) and other benzanilides (Gowda *et al.*, 2007a,b, 2008a,b). The bond parameters in N24DCPBA are similar to those in NPBA, N2CPBA, N4CPBA, N23DCPBA, N26DCPBA and other benzanilides. The amide group –NHCO– forms the dihedral angle of 33.0 (2)° with the benzoyl ring, while the benzoyl and aniline rings are almost coplanar, with the dihedral angle of 2.6 (2)°). Part of the crystal structure of the title compound with infinite molecular chains running in the [010] direction is shown in Fig. 2. The chains are generated by N—H···O(i) hydrogen bonds (Table 1) [symmetry operation (i): *x*,*y* - 1,*z*].

### Experimental

The title compound was prepared according to the literature method (Gowda *et al.*, 2003). The purity of the compound was checked by determining its melting point. It was characterized by recording its infrared and NMR spectra. Single crystals of the title compound were obtained from an ethanolic solution and used for X-ray diffraction studies at room temperature.

### Refinement

H atoms attached to C atoms were placed in calculated positions and subsequently treated as riding with C–H distance 0.93 Å. H atom of the amide group was refined with the N—H distance restrained to 0.81 (2) Å. The *U*_iso_(H) values were set at 1.2 *U*_eq_(C,*N*).

### Crystal data

**Table:**

C_13_H_9_Cl_2_NO	*F*_000_ = 544
*M**_r_* = 266.11	*D*_x_ = 1.446 Mg m^−^^3^
Monoclinic, *P*2_1_/*c*	Mo *K*α radiation λ = 0.71073 Å
Hall symbol: -P 2ybc	Cell parameters from 2675 reflections
*a* = 11.7388 (6) Å	θ = 3.5–29.1º
*b* = 4.7475 (2) Å	µ = 0.51 mm^−^^1^
*c* = 22.8630 (11) Å	*T* = 295 (2) K
β = 106.360 (4)º	Needle, colorless
*V* = 1222.56 (10) Å^3^	0.33 × 0.06 × 0.03 mm
*Z* = 4	

### Data collection

**Table:**

Oxford Diffraction Xcalibur diffractometer	2311 independent reflections
Monochromator: graphite	1209 reflections with *I* > 2σ(*I*)
Detector resolution: 10.434 pixels mm^-1^	*R*_int_ = 0.060
*T* = 295(2) K	θ_max_ = 25.7º
ω scans with κ offsets	θ_min_ = 5.1º
Absorption correction: analytical[CrysAlis RED (Oxford Diffraction, 2007), based on expressions derived by Clark & Reid (1995)]	*h* = −14→14
*T*_min_ = 0.905, *T*_max_ = 0.987	*k* = −5→5
11465 measured reflections	*l* = −27→27

### Refinement

**Table:**

Refinement on *F*^2^	Secondary atom site location: difference Fourier map
Least-squares matrix: full	Hydrogen site location: inferred from neighbouring sites
*R*[*F*^2^ > 2σ(*F*^2^)] = 0.037	H atoms treated by a mixture of independent and constrained refinement
*wR*(*F*^2^) = 0.089	[exp(3(sinθ/λ)^2^)]/ [σ^2^(*F*_o_^2^) + (0.0389*P*)^2^] where *P* = 0.33333*F*_o_^2^ + 0.66667*F*_c_^2^
*S* = 1.06	(Δ/σ)_max_ = 0.002
2311 reflections	Δρ_max_ = 0.21 e Å^−^^3^
157 parameters	Δρ_min_ = −0.16 e Å^−^^3^
1 restraint	Extinction correction: none
Primary atom site location: structure-invariant direct methods	

### Special details

**Table:**

Geometry. All e.s.d.'s (except the e.s.d. in the dihedral angle between two l.s. planes) are estimated using the full covariance matrix. The cell e.s.d.'s are taken into account individually in the estimation of e.s.d.'s in distances, angles and torsion angles; correlations between e.s.d.'s in cell parameters are only used when they are defined by crystal symmetry. An approximate (isotropic) treatment of cell e.s.d.'s is used for estimating e.s.d.'s involving l.s. planes.
Refinement. Refinement of *F*^2^ against ALL reflections. The weighted *R*-factor *wR* and goodness of fit *S* are based on *F*^2^, conventional *R*-factors *R* are based on *F*, with *F* set to zero for negative *F*^2^. The threshold expression of *F*^2^ > σ(*F*^2^) is used only for calculating *R*-factors(gt) *etc*. and is not relevant to the choice of reflections for refinement. *R*-factors based on *F*^2^ are statistically about twice as large as those based on *F*, and *R*- factors based on ALL data will be even larger.

### Fractional atomic coordinates and isotropic or equivalent isotropic displacement parameters (Å^2^)

**Table:**

	*x*	*y*	*z*	*U*_iso_*/*U*_eq_	
C1	1.0522 (2)	0.6218 (5)	0.12848 (10)	0.0496 (6)	
C2	1.1434 (2)	0.5105 (5)	0.18272 (10)	0.0528 (6)	
C3	1.2566 (3)	0.6149 (6)	0.19551 (12)	0.0702 (7)	
H3	1.2743	0.7524	0.1704	0.084*	
C4	1.3453 (3)	0.5182 (7)	0.24537 (14)	0.0849 (9)	
H4	1.4223	0.5874	0.2533	0.102*	
C5	1.3184 (4)	0.3218 (8)	0.28242 (14)	0.0893 (10)	
H5	1.3774	0.2567	0.316	0.107*	
C6	1.2056 (4)	0.2184 (6)	0.27103 (12)	0.0877 (10)	
H6	1.1882	0.0849	0.297	0.105*	
C7	1.1172 (3)	0.3122 (5)	0.22083 (11)	0.0677 (7)	
H7	1.0405	0.2413	0.213	0.081*	
C8	0.8686 (2)	0.4974 (4)	0.04960 (9)	0.0457 (6)	
C9	0.8747 (2)	0.6864 (4)	0.00429 (10)	0.0518 (6)	
H9	0.9462	0.7762	0.0064	0.062*	
C10	0.7767 (2)	0.7427 (5)	−0.04370 (10)	0.0597 (7)	
H10	0.7814	0.8708	−0.0737	0.072*	
C11	0.6721 (2)	0.6086 (6)	−0.04695 (11)	0.0611 (7)	
C12	0.6630 (2)	0.4146 (5)	−0.00389 (11)	0.0610 (7)	
H12	0.5919	0.3208	−0.0071	0.073*	
C13	0.7617 (2)	0.3632 (5)	0.04400 (10)	0.0510 (6)	
N1	0.96721 (19)	0.4383 (4)	0.09966 (8)	0.0513 (5)	
H1N	0.972 (2)	0.276 (4)	0.1100 (10)	0.062*	
O1	1.05520 (15)	0.8645 (3)	0.11131 (7)	0.0687 (5)	
Cl1	0.75028 (6)	0.12644 (14)	0.09989 (3)	0.0705 (2)	
Cl2	0.54660 (7)	0.6883 (2)	−0.10654 (3)	0.1010 (3)	

### Atomic displacement parameters (Å^2^)

**Table:**

	*U*^11^	*U*^22^	*U*^33^	*U*^12^	*U*^13^	*U*^23^
C1	0.0601 (15)	0.0357 (14)	0.0511 (13)	0.0050 (13)	0.0128 (12)	−0.0020 (11)
C2	0.0646 (18)	0.0412 (13)	0.0492 (13)	0.0076 (13)	0.0102 (12)	−0.0042 (11)
C3	0.0695 (19)	0.0686 (18)	0.0653 (17)	0.0051 (16)	0.0073 (14)	0.0003 (13)
C4	0.071 (2)	0.099 (2)	0.073 (2)	0.0125 (19)	0.0002 (17)	−0.0152 (19)
C5	0.100 (3)	0.089 (2)	0.0600 (19)	0.036 (2)	−0.0077 (18)	−0.0101 (17)
C6	0.131 (3)	0.072 (2)	0.0491 (16)	0.016 (2)	0.0083 (18)	0.0054 (14)
C7	0.093 (2)	0.0550 (17)	0.0504 (14)	0.0032 (15)	0.0121 (14)	0.0012 (12)
C8	0.0541 (16)	0.0349 (12)	0.0482 (13)	0.0057 (12)	0.0146 (11)	−0.0021 (11)
C9	0.0556 (15)	0.0437 (14)	0.0557 (14)	0.0016 (12)	0.0150 (12)	0.0043 (11)
C10	0.0700 (19)	0.0588 (15)	0.0510 (14)	0.0114 (14)	0.0184 (13)	0.0099 (12)
C11	0.0547 (17)	0.0721 (17)	0.0526 (14)	0.0143 (15)	0.0086 (12)	−0.0020 (14)
C12	0.0549 (16)	0.0659 (17)	0.0645 (16)	−0.0021 (13)	0.0205 (13)	−0.0069 (14)
C13	0.0566 (16)	0.0454 (13)	0.0541 (14)	0.0010 (13)	0.0204 (12)	−0.0024 (11)
N1	0.0636 (13)	0.0342 (11)	0.0531 (11)	0.0008 (11)	0.0117 (10)	0.0065 (9)
O1	0.0800 (12)	0.0347 (10)	0.0776 (11)	−0.0009 (9)	−0.0003 (9)	0.0063 (8)
Cl1	0.0783 (5)	0.0633 (4)	0.0772 (4)	−0.0034 (4)	0.0339 (4)	0.0109 (3)
Cl2	0.0706 (5)	0.1426 (8)	0.0752 (5)	0.0183 (5)	−0.0033 (4)	0.0119 (4)

### Geometric parameters (Å, °)

**Table:**

C1—O1	1.221 (2)	C8—C13	1.380 (3)
C1—N1	1.348 (3)	C8—C9	1.388 (3)
C1—C2	1.488 (3)	C8—N1	1.408 (3)
C2—C3	1.371 (3)	C9—C10	1.374 (3)
C2—C7	1.375 (3)	C9—H9	0.93
C3—C4	1.387 (4)	C10—C11	1.366 (3)
C3—H3	0.93	C10—H10	0.93
C4—C5	1.356 (4)	C11—C12	1.374 (3)
C4—H4	0.93	C11—Cl2	1.743 (2)
C5—C6	1.366 (5)	C12—C13	1.374 (3)
C5—H5	0.93	C12—H12	0.93
C6—C7	1.387 (4)	C13—Cl1	1.735 (2)
C6—H6	0.93	N1—H1N	0.805 (16)
C7—H7	0.93		
			
O1—C1—N1	122.5 (2)	C13—C8—C9	117.8 (2)
O1—C1—C2	121.6 (2)	C13—C8—N1	120.1 (2)
N1—C1—C2	115.9 (2)	C9—C8—N1	122.2 (2)
C3—C2—C7	119.3 (2)	C10—C9—C8	120.9 (2)
C3—C2—C1	118.4 (2)	C10—C9—H9	119.5
C7—C2—C1	122.3 (2)	C8—C9—H9	119.5
C2—C3—C4	120.9 (3)	C11—C10—C9	119.4 (2)
C2—C3—H3	119.6	C11—C10—H10	120.3
C4—C3—H3	119.6	C9—C10—H10	120.3
C5—C4—C3	119.2 (3)	C10—C11—C12	121.6 (2)
C5—C4—H4	120.4	C10—C11—Cl2	119.3 (2)
C3—C4—H4	120.4	C12—C11—Cl2	119.1 (2)
C4—C5—C6	120.8 (3)	C13—C12—C11	118.1 (2)
C4—C5—H5	119.6	C13—C12—H12	120.9
C6—C5—H5	119.6	C11—C12—H12	120.9
C5—C6—C7	120.1 (3)	C12—C13—C8	122.2 (2)
C5—C6—H6	119.9	C12—C13—Cl1	118.62 (19)
C7—C6—H6	119.9	C8—C13—Cl1	119.18 (17)
C2—C7—C6	119.7 (3)	C1—N1—C8	126.45 (18)
C2—C7—H7	120.2	C1—N1—H1N	119.5 (18)
C6—C7—H7	120.2	C8—N1—H1N	114.0 (18)
			
O1—C1—C2—C3	−31.9 (3)	C9—C10—C11—C12	−1.2 (4)
N1—C1—C2—C3	147.8 (2)	C9—C10—C11—Cl2	178.01 (17)
O1—C1—C2—C7	146.7 (2)	C10—C11—C12—C13	1.7 (4)
N1—C1—C2—C7	−33.6 (3)	Cl2—C11—C12—C13	−177.46 (18)
C7—C2—C3—C4	1.6 (4)	C11—C12—C13—C8	−0.6 (3)
C1—C2—C3—C4	−179.8 (2)	C11—C12—C13—Cl1	178.13 (19)
C2—C3—C4—C5	−1.3 (4)	C9—C8—C13—C12	−1.0 (3)
C3—C4—C5—C6	0.2 (4)	N1—C8—C13—C12	179.8 (2)
C4—C5—C6—C7	0.5 (4)	C9—C8—C13—Cl1	−179.73 (16)
C3—C2—C7—C6	−0.8 (4)	N1—C8—C13—Cl1	1.0 (3)
C1—C2—C7—C6	−179.4 (2)	O1—C1—N1—C8	−3.9 (4)
C5—C6—C7—C2	−0.2 (4)	C2—C1—N1—C8	176.4 (2)
C13—C8—C9—C10	1.6 (3)	C13—C8—N1—C1	−145.5 (2)
N1—C8—C9—C10	−179.2 (2)	C9—C8—N1—C1	35.3 (3)
C8—C9—C10—C11	−0.5 (3)		

### Hydrogen-bond geometry (Å, °)

**Table:**

*D*—H···*A*	*D*—H	H···*A*	*D*···*A*	*D*—H···*A*
N1—H1N···O1^i^	0.805 (16)	2.178 (19)	2.899 (2)	149 (2)

Symmetry codes: (i) *x*, *y*−1, *z*.

## References

[bb1] Brandenburg, K. (2002). *DIAMOND* Crystal Impact GbR, Bonn, Germany.

[bb2] Clark, R. C. & Reid, J. S. (1995). *Acta Cryst.* A**51**, 887–897.

[bb3] Farrugia, L. J. (1997). *J. Appl. Cryst.* **30**, 565.

[bb4] Farrugia, L. J. (1999). *J. Appl. Cryst.* **32**, 837–838.

[bb5] Gowda, B. T., Jyothi, K., Paulus, H. & Fuess, H. (2003). *Z. Naturforsch. Teil A*, **58**, 225–230.

[bb6] Gowda, B. T., Sowmya, B. P., Kožíšek, J., Tokarčík, M. & Fuess, H. (2007*a*). *Acta Cryst.* E**63**, o2906. 10.1107/S1600536807066937 PMC2915381 21200902

[bb7] Gowda, B. T., Sowmya, B. P., Tokarčík, M., Kožíšek, J. & Fuess, H. (2007b). *Acta Cryst.* E**63**, o3326. 10.1107/S1600536807066937 PMC2915381 21200902

[bb8] Gowda, B. T., Tokarčík, M., Kožíšek, J., Sowmya, B. P. & Fuess, H. (2008*a*). *Acta Cryst.* E**64**, o540. 10.1107/S160053680800305X PMC2960174 21201559

[bb9] Gowda, B. T., Tokarčík, M., Kožíšek, J., Sowmya, B. P. & Fuess, H. (2008*b*). *Acta Cryst.* E**64**, o769. 10.1107/S1600536808008155 PMC2961007 21202156

[bb10] Oxford Diffraction (2007). *CrysAlis CCD* and *CrysAlis RED* Oxford Diffraction Ltd, Abingdon, Oxfordshire, England.

[bb11] Sheldrick, G. M. (2008). *Acta Cryst.* A**64**, 112–122. 10.1107/S0108767307043930 18156677

[bb12] Spek, A. L. (2003). *J. Appl. Cryst.* **36**, 7–13.

